# Decline of Birds in a Human Modified Coastal Dune Forest Landscape in South Africa

**DOI:** 10.1371/journal.pone.0016176

**Published:** 2011-01-13

**Authors:** Morgan J. Trimble, Rudi J. van Aarde

**Affiliations:** Conservation Ecology Research Unit, Department of Zoology and Entomology, University of Pretoria, Pretoria, South Africa; University of California, Berkeley, United States of America

## Abstract

Previous studies demonstrate that old-growth forest remnants and vegetation regenerating after anthropogenic disturbance provide habitat for birds in a human modified coastal dune forest landscape in northern KwaZulu-Natal, South Africa. However, occurrence does not ensure persistence. Based on a 13-year monitoring database we calculated population trends for 37 bird species and general trends in overall bird density in different vegetation types. We evaluated species' characteristics as covariates of population trend and assessed changes in rainfall and proportional area and survey coverage per vegetation type. 76% of species assessed have declined, 57% significantly so at an average rate of 13.9% per year. Overall, bird density has fallen at 12.2% per year across old-growth forest and woody regenerating vegetation types. Changes in proportional area and coverage per vegetation type may partly explain trends for a few species but are unlikely to account for most. Below average rainfall may have contributed to bird declines. However, other possibilities warrant further investigation. Species with larger range extents tended to decline more sharply than did others, and these species may be responding to environmental changes on a broader geographical scale. Our results cast doubt on the future persistence of birds in this human modified landscape. More research is needed to elucidate the mechanisms driving population decline in the study area and to investigate whether the declines identified here are more widespread across the region and perhaps the continent.

## Introduction

Coastal dune forest is one of South Africa's rarest vegetation types; restricted to the eastern coast, it covers less than 1000 km^2^. It is also biogeographically important, and occurs within the Maputaland Center of endemism [1] and the Maputaland-Pondoland-Albany biodiversity hotspot [2], [3]. While South African coastal dune forest is relatively well protected with 9.51% conserved, 43% has been transformed [4]. The coastal location on the Indian Ocean accounts for the biggest threats to coastal dune forests—holiday resort expansion, dune mining, and firewood collection and clearing for agriculture by local communities [4]. Additionally, the narrowness and linear nature of the coastal dune forest belt might make it particularly susceptible to edge effects, fragmentation, and isolation [5].

Forest conservation depends on maintaining both the land covered by forests and the ecological processes necessary for plant regeneration and gene flow [4]. Thus, isolated stands of protected coastal dune forests may be insufficient for their long-term conservation [4] because dispersal ability of many tree species is constrained by distance between forest patches [6]. Due to their vagility and role in seed dispersal [7], birds may enhance connectivity of coastal dune forest fragments (see [6]). Thus, promoting persistence of coastal dune forest birds beyond protected areas may be important for both bird and forest conservation and is in line with recent shifts in conservation ideology from a strictly protected area based approach to a wider consideration of biodiversity in human modified landscapes [8], [9]. Land-use options that incorporate coastal dune forest elements such as remnant forest patches in agricultural landscapes or active regeneration after anthropogenic disturbances may allow bird populations to persist beyond protected areas. This may be the case in South Africa's northern coastal dune forests.

North of Richards Bay, on the coast of KwaZulu-Natal province, opencast surface mining of sand dunes has occurred since 1977 and has been followed by an active rehabilitation program to return indigenous coastal dune vegetation to one third of the mined area (see [10] for program description). Our earlier work showed that, with age, bird communities in the successional sere of known-aged regenerating sites become more similar to that of old-growth coastal dune forest [10]–[14]. These observations suggest that post-mining regenerating forests and old-growth forest remnants provide refuge for coastal dune forest birds beyond protected areas—*e.g.* the Richards Bay Game Reserve ∼20 km to the southwest and the iSimangaliso Wetland Park and World Heritage Site ∼5 km to the northeast. However, these studies were based on snapshots of bird occurrence, and occurrence of species does not ensure their persistence (see [9], [15]). Assessing changes in population size over time is a step towards understanding the processes (*e.g.* survival, fecundity, and dispersal (see [15], [16])) that affect patterns of species occurrence and persistence in human modified landscapes.

Based on 13 years of quantitative monitoring of forest birds, we calculated population trends for birds found commonly in old-growth coastal dune forest and woody regenerating vegetation types. We also calculated general trends of overall bird densities over time in old-growth forest and woody regenerating vegetation types. We investigated how species' characteristics known to be associated with extinction proneness of forest birds—*i.e.* clutch size, habitat affinity, diet, tolerance of human modified landscapes, and range extent (see [17] and references therein)—related to population trend and assessed changes in rainfall, proportional area of vegetation types, and survey coverage per vegetation type as possible determinants of population and general trends.

## Methods

### Bird Data

We used data collected as part of a long-term monitoring program designed to assess the success of coastal dune forest rehabilitation after dune mining (see [10] and [13] for a description of the program and map of the study area). Between 1997 and 2009, birds were surveyed via transect counts in 9 survey years at two relatively pristine old-growth coastal dune forest sites and nine regenerating forest sites of known age (Table S1) within a mining lease area maintained by Richards Bay Minerals (RBM). Forest regeneration in the area follows a trajectory of vegetation types from grassland (∼1–5 years old), to thicket (∼6–12 years old), to an early woodland stage dominated by *Acacia karroo* (∼12–20 years old), to a late woodland stage in which *Acacia karroo* individuals have senesced and been replaced by coastal dune forest trees (∼20–35 years old)—see [6], [10]. Experienced observers walked 250–500 m transects randomly located at least 200 m apart within vegetation types (Table S1) and recorded birds seen and distance from the transect. In most years, exact distances were recorded up to 60 m but in 1997 and 2006, distance intervals were used with cut points 2, 5, 10, 20, and 40 m and 5, 10, 15, 20, 25, and 30 m respectively. Birds flying over the canopy and all raptors, aerial feeders, and nocturnal birds were excluded. All surveys were conducted in the early morning under favorable weather conditions and took place between November and February.

Throughout the study period, 102 species were represented in 7890 sightings. We narrowed the species list to focal species typical of old-growth forest and the woody regenerating vegetation types—thicket, early woodland, and late woodland. To do this, we assessed the affinity of each species towards different vegetation types. For each species, we calculated the overall number of sightings/km of transect in each vegetation type—grassland, thicket, early woodland, late woodland, and old-growth forest. Twenty-seven species had ≥60% of their sightings/km in grassland, and we excluded all but two of these species from further analyses. We retained Red-eyed Dove and Yellow-eyed Canary because, although the majority of sightings were in grassland, they were also quite common in old-growth forest with >20% sightings/km. We also excluded Lesser-masked Weaver *Ploceus intermedius* (predominantly found in thicket) from further analysis because observers in different years variably distinguished between Lesser-masked and other similar looking weavers predominantly found in grassland (*i.e.* Village Weaver *Ploceus cucullatus* and Yellow Weaver *Ploceus subaureus*). Thus, 6868 sightings of 76 species were retained for further analysis. We separated these species into two groups—39 relatively rare species (recorded ≤20 times throughout the study period) and 37 relatively common species (recorded >20 times). Common and scientific names are provided in Table 1 for relatively common species and Table S2 for relatively rare species.

**Table 1:** Population trends and covariates for relatively common species.

Common name	Scientific name	Pool	Trend	SE	Range(km^2^)	Predominant habitat	OG affinity
Black-bellied Starling	*Lamprotornis corruscus*	C	0.104	0.063	350000	OG (0.54)	0.54
Ashy Flycatcher	*Muscicapa caerulescens*	B	−0.171*	0.055	7700000	EW (0.47)	0.21
Black-backed Puffback	*Dryoscopus cubla*	C	−0.083*	0.037	5400000	OG (0.34)	0.34
Black-throated Wattle-Eye	*Platysteira peltata*	B	0.027	0.048	3100000	OG (0.81)	0.81
Blue-mantled Crested-Flycatcher	*Trochocercus cyanomelas*	A	−0.077	0.073	1200000	OG (1)	1.00
Brown-hooded Kingfisher	*Halcyon albiventris*	C	−0.008	0.067	3800000	EW (0.39)	0.08
Burchell's Coucal	*Centropus burchellii*	C	−0.153*	0.067	5000000	OG (0.42)	0.42
Cape White-eye	*Zosterops virens*	B	−0.090*	0.042	1300000	OG (0.38)	0.23
Collared Sunbird	*Hedydipna collaris*	B	−0.132*	0.051	5500000	OG (0.52)	0.52
Dark-backed Weaver	*Ploceus bicolor*	C	0.051	0.027	1100000	OG (0.33)	0.33
Dark-capped Bulbul	*Pycnonotus tricolor*	C	−0.126*	0.029	19000000	G (0.25)	0.23
Eastern Nicator	*Nicator gularis*	C	−0.098	0.071	4000000	OG (0.38)	0.38
Fork-tailed Drongo	*Dicrurus adsimilis*	C	−0.243*	0.071	14000000	EW (0.67)	0.09
Golden-tailed Woodpecker	*Coampethera abingoni*	C	0.377*	0.104	3880000	LW (0.56)	0.27
Green Malkoha	*Ceuthmochares aereus*	C	−0.141	0.091	5400000	OG (0.82)	0.82
Green-backed Camaroptera	*Camaroptera brachyura*	A	−0.144*	0.029	16000000	EW (0.3)	0.18
Grey Sunbird	*Cyanomitra veroxii*	B	−0.160*	0.064	170000	OG (0.47)	0.47
Hadeda Ibis	*Bostrychia hagedash*	C	0.270	0.153	16000000	G (0.32)	0.25
Lemon Dove	*Aplopelia larvata*	A	0.082	0.089	2000000	OG (1)	1.00
Livingstone's Turaco	*Tauraco livingstonii*	C	−0.154*	0.042	5000000	OG (1)	1.00
Olive Sunbird	*Cyanomitra olivacea*	B	−0.127*	0.029	570000	OG (0.45)	0.45
Red-capped Robin Chat	*Cossypha natalensis*	A	−0.137*	0.031	3600000	OG (0.58)	0.58
Red-eyed Dove	*Streptopelia semitorquata*	C	−0.197	0.182	10000000	G (0.68)	0.26
Rudd's Apalis	*Apalis ruddi*	B	−0.116*	0.021	76000	T (0.46)	0.20
Sombre Greenbul	*Andropadus importunes*	C	−0.105*	0.035	1200000	OG (0.52)	0.52
Southern Boubou	*Laniarius ferrugineus*	B	−0.153*	0.046	580000	OG (0.7)	0.70
Square-tailed Drongo	*Dicrurus ludwigii*	C	−0.034	0.029	4300000	OG (0.37)	0.37
Tambourine Dove	*Turtur tympanistria*	A	0.018	0.057	7400000	OG (0.41)	0.41
Tawny-flanked Prinia	*Prinia subflava*	B	−0.202*	0.042	14000000	G (0.44)	0.12
Terrestrial Brownbul	*Phyllastrephus terrestris*	A	−0.181*	0.073	2400000	OG (0.9)	0.90
Trumpeter Hornbill	*Bycanistes bucinator*	C	0.045	0.098	2900000	OG (0.96)	0.96
White-browed Robin-Chat	*Cossypha heuglini*	C	−0.326*	0.058	8800000	T (0.49)	0.00
White-eared Barbet	*Stactolaema leucotis*	C	0.005	0.071	710000	OG (0.59)	0.59
Yellow-bellied Greenbul	*Chlorocichla flaviventris*	C	−0.095*	0.024	3800000	OG (0.49)	0.49
Yellow-breasted Apalis	*Apalis Favida*	B	−0.133*	0.031	5600000	EW (0.38)	0.13
Yellow-fronted Canary	*Crithagra mozambicus*	C	−0.207	0.108	9500000	G (0.6)	0.22
Yellow-rumped Tinkerbird	*Pogoniulus bilineatus*	B	−0.142*	0.063	6600000	OG (0.75)	0.75

Species names follow [29]. Pool codes are A = furtive species, B = intermediate, C = conspicuous.

*indicates statistically significant trends. Predominant habitat is the vegetation type in which a species has the greatest proportion of sightings/km, and the proportion is given in parentheses. Vegetation type abbreviations as follows: OG = old-growth coastal dune forest, LW = late woodland, EW = early woodland, T = thicket, G = grassland. OG affinity is the proportion of sighting/km in old-growth forest.

To our knowledge, this is one of few long-term quantitative bird monitoring datasets for Africa. However, some aspects of the survey methodology might introduce bias. Differences in observers and vegetation types may lead to variation in the probability of detecting birds, which could bias inferences on the change in bird densities over time [18]. We used distance sampling techniques to account for variability in detection probability to generate more reliable density estimates than unadjusted counts provide. Distance sampling relies on creating a detection function of the frequency of observations on distance from the transect line to estimate the average detection probability 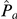 of observing a bird given it is within the truncation point *w* of the line transect [19].

To calculate reliable detection functions, 60–80 observations are necessary [19], but in our study, most species were recorded far less often than 60 times per year. Similarly detectable species can be grouped together to achieve sufficient sample size to calculate a common detection function [19]. Thus, we grouped the 37 relatively common species (those recorded >20 times) into three species pools: pool A—furtive species generally seen very close to the transect line, pool B—species that are intermediately visible, and pool C—conspicuous species frequently seen far from the transect line (Table 1). For each of these species pools, we used the Multiple-Covariate Distance Sampling (MCDS) engine in the program DISTANCE, version 6.0 [20] to fit four detection function models for each year: a half-normal key model, a hazard-rate key model, and each with vegetation type as a factor covariate. Additionally, for 2007–2009 when two observers conducted surveys, we also fitted a half-normal and hazard-rate model with observer as a factor covariate and with both observer and vegetation type as factor covariates. Estimating a single detection function per year by pooling over vegetation types and observer differences should give adequate global estimates due to the pooling robustness property of distance sampling, but including these variables in MCDS can lead to increased estimate precision [18]. We did not use adjustment terms in the models to avoid implausible, non-monotonic function results [18]. To achieve adequate model fit and estimator robustness, we set distance intervals and truncation points to accommodate characteristics of species pools (*e.g.* shorter truncation point for furtive species), occasional issues with distance heaping and evasive movement of birds away from the transect line, and distance data collection intervals for 1997 and 2006. Models were post-stratified by species, but estimates were made at the global level, meaning that species in the same pool had a common detection function per year. We selected the best model per year based on AIC and extracted an estimate for 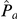
and its SE.

We assessed support for our assumption that species within each pool shared similar detectability by fitting detection functions to the total dataset (years pooled). We used the MCDS engine to fit for each species pool half-normal and hazard-rate key models, each with vegetation type as a factor covariate, each with observer as a factor covariate, and each with species as a factor covariate. We then compared the models with AIC to assess whether pooling species was a reasonable assumption.

We were also interested in annual estimates specific to vegetation types. We modeled the per year, per vegetation type detection functions for birds in general (all 76 species pooled). We used the MCDS engine to fit for each year a half-normal key model and a hazard-rate key model and, for 2007–2009, each with observer as a factor covariate. Again, we did not use further adjustment terms and selected the best model per year based on AIC. Models were post-stratified by vegetation type with estimates made at the vegetation type stratum level. This generated an estimate for 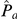 and its SE of birds in general per vegetation type per year.

### Trends and Determinants

We assessed population trends over time for the 37 species recorded >20 times. We used quasi-Poisson generalized linear modeling (GLM) with log-link function and standard errors corrected for over-dispersion [21] and detection probability incorporated as an offset term [22]. We fitted the model *n_t,s_* = exp(log*_e_*(2*L_t_w_t_* 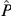 *_a,p,t_*)+*β_0_*+ *β_1_t*)+*ε_t_* where *n_t,s_* is the number of birds of species *s* counted in year *t*, *L_t_* is the line length surveyed at time *t*, *w_t_* is the truncation distance, 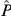 *_a,p,t_* is the estimated mean probability of detection for species in pool *p* in the covered region *a* in year *t,* and log*_e_*(2*L_t_w_t_* 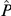 *_a,p,t_*) is the offset term (modified from [22]). In GLM, offsets are assumed known, but 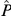 *_a,p,t_* is an estimate [22]. To account for uncertainty in the estimate of 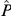 *_a,p,t_*, we randomly resampled each estimate 999 times from a lognormal distribution and refit the GLM to each resample. We then estimated population trend and SE as the mean slope parameter and SE estimates from 999 fitted GLM's for each species. Population trends were deemed significant when population trend ± 1.96 SE did not include 0. Percent change per year was calculated as (exp(population trend) – 1)*100.

We followed the same procedure to estimate general trends in bird density in each vegetation type by substituting into the GLM equation *n_t,v_*, number of bird sightings per vegetation type *v* in year *t*, and 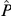 *_a,p,t_* the estimated mean probability of detection of birds in vegetation type *v* in the covered region *a* in year *t*. Subsequently, we checked for significant differences of slopes and intercepts between vegetation types with a GLM of *n_t,v_* on *t* with an offset as described previously, a categorical variable of vegetation type *v*, and an interaction term between *t* and *v*. Significance of the interaction term indicates significantly different slopes.

We only calculated population trends for species recorded >20 times. To infer what might be happening to the 39 relatively rare species, we assessed how commonness influenced population trend estimates. To do this, we regressed population trend estimate and SE on log*_e_* of the cumulative number of sightings per species throughout the study period.

Variables that are intrinsic to species might explain variation in population trends. These include habitat affinity [23], mean clutch size and bird weight (proxies for life history characteristics [24]), diet [25], [26], tolerance of human modified landscapes [27], and range extent [28]. We assigned habitat affinity as a categorical variable—predominant habitat—based on the vegetation type in which a species had the highest proportion of sightings/km. We also quantified affinity for old-growth forest as the proportion of sightings/km in old-growth forest. We sourced clutch size, weight, diet, and tolerance data for relevant species [29]. Based on the predominant food items listed, we distinguished three diet preference classes: insects and other invertebrates; plant material; and omnivorous/carnivorous. We considered species listed to occur in gardens, parks, plantations, and cultivated areas tolerant of human modified landscapes while others were deemed intolerant. Finally, we noted the extent of each species' resident range [30]. We assessed the relationship between population trend and range extent, affinity for old-growth forest, clutch size, and weight with linear regression. We used t-tests to compare population trends between species with predominant habitat in old-growth forest and those with predominant habitat in one of the regenerating vegetation types and between species that are tolerant and intolerant of human modified landscapes. We used ANOVA to compare population trends between the three diet preference classes. Some caution is required in comparing population trends among species because pooling species to calculate detection functions means that annual density estimates from the same pool are not independent [19]. Therefore, species pooling could influence trend estimates. Thus, we used ANOVA to compare population trend estimates between the three species pools.

We also assessed factors that might influence both population trends and general trends —changes in rainfall [31], area of each vegetation type [32], [33], and transect coverage per vegetation type. We quantified mean annual rainfall as the residual cumulative annual rainfall (January–December) compared to the long-term mean annual rainfall (1977–2009). Rainfall data (provided by RBM) was unavailable for 2008. Proportional area of each vegetation type was calculated based on the area and age of each site in each year, and we assessed change over time with linear regression. Coverage per year was calculated as the proportion of km's of transect in each vegetation type per year. We assessed whether changes in coverage have generally matched changes in area by regressing proportional coverage divided by proportional area on year for each vegetation type.

## Results

### Habitat Affinity

Of the 37 commonly observed species, 3 were only recorded in old-growth forest and 4 more had ≥80% of their sightings/km in old-growth forest. The majority of species (24) were often recorded in old-growth forest (≥20%, <80% sightings/km) but also frequently seen in regenerating vegetation types. Six species were rarely seen in old-growth forest (<20% sightings/km) including one species never recorded there (Table 1). Habitat affinities should be taken as an index comparable among species rather than as an absolute measure of species' habitat preferences because sightings/km were not corrected for variability in detection probability among vegetation types. We did not assess the habitat affinities of the 39 rarely observed species (those recorded ≤20 times) because so few sightings are unlikely to be representative of the species' occurrence in different vegetation types.

### Distance Sampling

We fitted detection functions for each of the three species pools in each year (Table 1, Table S3). Detection probability varied among species pools with furtive species being the least detectable and conspicuous species the most, although estimates are not directly comparable due to variability in truncation distance (Table S3). Our assumption of relatively similar bird detectability within pools was supported, and models with species as a covariate were the least likely compared to models with a vegetation type covariate, an observer covariate and no covariate for all three species pools (Table S4). We also fitted detection functions for birds in general (76 species pooled) for each vegetation type in each year (Table S5). As expected, detection probability was generally high in early and late woodland, low in thicket, and intermediate in old-growth forest. There were too few observations in grassland to fit per year detection functions.

### Population Trends and Determinants

**Figure 1:**
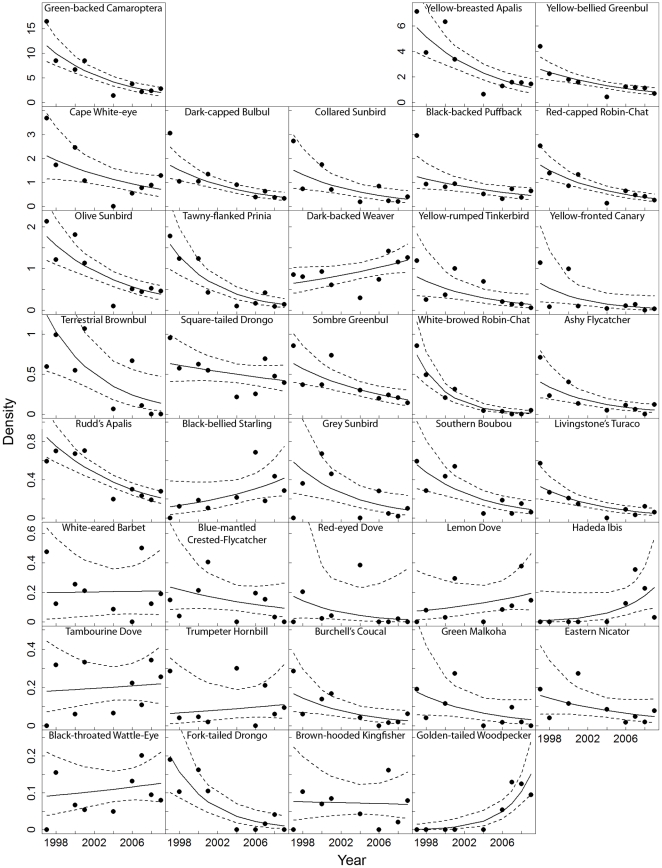
Population trends. Change in density/ha over time for relatively common species with fitted GLM trend line and 95% CI (stippled lines) from the original offset estimate. Density was estimated by *n_t,s_*/2*L_t_w_t_* 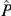 *_a,p,t_* where *n_t,s_* is the number of sightings per species per year, 2*L_t_w_t_* is the area of transect coverage in hectares and 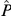 *_a,p,t_* is the detection probability over the area covered per pool per year. See Table 1 for trend estimates and SE's calculated based on 999 resamples of 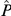 *_a,p,t_*.

We estimated population trends for the 37 relatively common species (recorded >20 times) (Table 1, Fig. 1). Twenty-eight of these species (76%) decreased, 21 significantly so at an average rate of 13.9% per year. Nine species (24%) increased but only one significantly so. Population trend estimates were not significantly related to the log*_e_* of the cumulative sightings/species (slope = −0.003, p = 0.88). However, as expected, SE of population trend estimates decreased with an increasing log*_e_* of cumulative sightings/species (slope = −0.02, r^2^ = 0.41, p<0.01).

Population trend estimates for 30 species were acceptably reliable (SE<0.08) for further analyses regarding the potential determinants of population trends. We investigated the relationship between population trends and characteristics of these species—range extent, affinity for old-growth forest, predominant habitat, clutch size, weight, predominant diet, and tolerance for human modified landscapes. Range extent was significantly related to population trend (slope = −7.63×10^−9^, r^2^ = 0.18, p<0.05) and was significantly correlated with affinity for old-growth forest (Pearson r = −0.46, p<0.05). However, affinity for old-growth forest was not significantly related to population trend (slope = −0.075, p = 0.23). Generally, species with larger ranges had lower population trends (*i.e.* more negative) and a lower affinity for old-growth forest. Species with predominant habitat among regenerating vegetation types had larger range extents than species with predominant habitat in old-growth forest (mean range extent per vegetation type: old-growth = 3.10×10^6^ km^2^, n = 20; regenerating = 9.03×10^6^ km^2^, n = 10; r^2^ = 0.33; p<0.01). Furthermore, species with predominant habitat among regenerating vegetation types had significantly lower population trends (*i.e.* more negative) than those with old-growth forest as predominant habitat (mean population trend per vegetation type: old-growth = −0.08, n = 20; regenerating = −0.16, n = 10; r^2^ = 0.16; p<0.05). Weight (slope = −2.5×10^−6^, p = 0.99), clutch size (slope = 0.016, p<0.57), predominant diet (mean population trend per diet class: insects = −0.12, n = 16; plants = −0.10, n = 10; omnivorous/carnivorous = −0.073, n = 4; p = 0.68), and tolerance for human modified landscapes (mean population trend per class: tolerant = −0.13, n = 13; intolerant = −0.09, n = 17; p = 0.30) were not significantly related to population trend. Furthermore, species pool was not significantly related to population trend (mean population trend per pool: pool A = −0.104, n = 5; pool B = −0.127, n = 11; pool C = −0.090, n = 14; p = 0.61).

**Figure 2:**
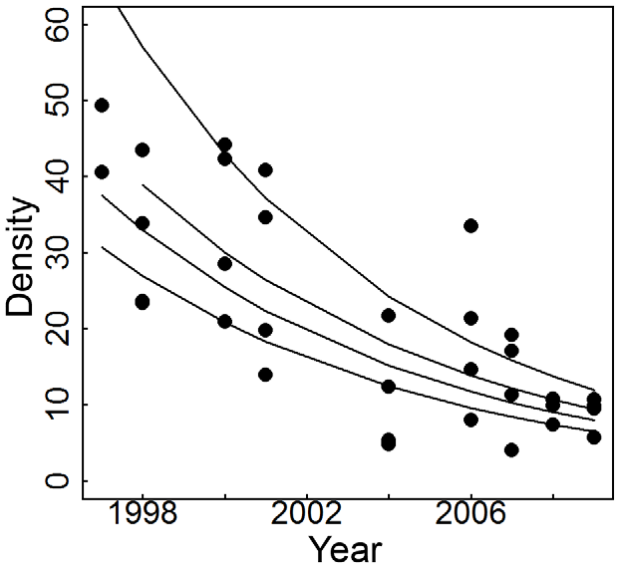
Vegetation type specific trends. Change in density/ha of birds in general over time in different vegetation types with fitted GLM trend lines of slope −0.13±0.01. Density was estimated by *n_t,v_*/2*L_t_w_t_* 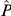 *_a,p,t_* where *n_t,v_* is the number of bird sightings per vegetation type per year, 2*L_t_w_t_* is the area of transect coverage in hectares and 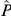 *_a,p,t_* is the detection probability over the area covered per vegetation type per year. Intercepts are significantly different and trend lines are for, from highest to lowest density, old-growth forest, late woodland, thicket, and early woodland.

We also assessed general trends of overall bird density (76 species pooled) in different vegetation types—thicket, early woodland, late woodland, and old-growth forest. Grassland had too few sightings/year to estimate detection functions. Birds declined significantly in early woodland, late woodland, and old-growth forest with mean general trend and SE from 999 detection probability resamples and GLM refittings of −0.13±0.03, −0.09±0.04, and −0.14±0.03 respectively. Birds also declined in thicket but not significantly so with mean general trend and SE  =  −0.15±0.10. However, general trends in different vegetation types did not differ significantly although the intercepts did. Thus, the overall general trend across old-growth, late woodland, early woodland, and thicket was −0.13±0.01 (Fig. 2).

We assessed changes in rainfall, area of vegetation types, and transect coverage per vegetation type over time as potential factors that could influence both population trends and general trends in overall bird density. Mean annual rainfall did not change significantly over time (slope = −62.10, p = 0.05). However, for 9 of 12 years for which we have rainfall data (1997–2009 excluding 2008 when data were unavailable), mean annual rainfall was lower than the long-term mean (Fig. S1). Furthermore, mean annual rainfall has been below the long-term mean every year since 2002. Proportional area of regenerating vegetation types changed over time as regenerating sites aged. Proportional area increased significantly over time for late woodland (slope = 0.019, r^2^ = 0.91, p<0.01) and thicket (slope = 0.005, r^2^ = 0.55, p<0.05) and decreased for early woodland (slope = −0.005, r^2^ = 0.52, p<0.05), while proportional area of grassland did not change significantly (slope = 0.001, p = 0.76). However, transect coverage per vegetation type, generally matched these changes with proportional coverage/proportional area per vegetation type not changing significantly over time for any vegetation type (old-growth forest: slope = −0.002, p = 0.86; late woodland: −0.024, p = 0.74; early woodland: slope = −0.033, p = 0.31; thicket: slope = −0.043, p = 0.08) except grassland (slope = −0.146, r^2^ = 0.58, p<0.05).

## Discussion

The birds inhabiting the old-growth coastal dune forests and coastal woody regenerating vegetation types (thicket, early woodland, and late woodland) have generally declined since 1997. Of the 37 relatively common species, 21 have declined significantly at rates between 7.9 and 27.8% per year while only one species has increased significantly. Furthermore, Rudd's Apalis, the only one of the four restricted-range bird species of the Maputaland Centre of endemism [3] to occur at our study site, has declined significantly at a rate of 10.9% per year. None of the species for which we assessed population trends are globally threatened [30], but they were, by necessity of the trend analysis procedure, relatively common in the study area. Species with reliable population trend estimates (SE<0.08) tended to be the most often recorded among the relatively common species because SE of population trend estimates decreased with increasing cumulative records per species. However, population trend estimate itself was not dependent on cumulative records per species, so there is no indication that populations of the 39 relatively rare species have fared better than the relatively common species.

Our earlier studies show that forest regeneration in the area results in increased bird species diversity with regeneration age, while overall density remains relatively stable [12] as the bird community undergoes a compositional shift from grassland and pioneer species to secondary forest species [11], [14]. Thus, from a site-specific perspective, a few species characteristic of early successional stages should decrease over time while many forest species increase as the regenerating vegetation becomes more similar to old-growth coastal dune forest. However, we took a study area wide view of population trends (necessitated by sample size requirements of distance sampling) rather than a site-specific approach. Therefore, successional changes in regenerating sites should not affect population trends unless area or transect coverage per vegetation type changes over time. While changes in area of vegetation types could result in real changes in population densities [32],[33], changes in coverage per vegetation type could generate false trends. Changes in coverage mirrored changes in area for all vegetation types except grassland, which became less well represented in sampling over time. Thus, population trend estimates for the birds found commonly in grasslands could have been negatively biased—primarily Red-eyed Dove, Yellow-fronted Canary, and Tawny-flanked Prinia with 68, 60, and 44% of their sightings/km in grassland respectively. Late woodland increased substantially in proportional area (0.02 per year), and the only bird to increase significantly, Golden-tailed Woodpecker, was also the only bird with predominant habitat in late woodland. While thicket increased significantly and early woodland decreased significantly in proportional area, the change was not substantial (−0.005 and +0.005 per year respectively).

Of the species' characteristics we assessed as potential determinants of population trends, only predominant habitat and range extent were related to population trend. Range extent was inversely proportional to population trend and to species' affinity for old-growth forest.

Additionally, species found predominantly in regenerating vegetation types had lower population trend estimates (*i.e.* more negative) and larger ranges than species found predominantly in old-growth forest. Species with large range extents tend to be generalists and are expected to have broad environmental tolerances [34], so specialists are generally more extinction prone [35]. Thus, it is surprising that species with large range extents tended to decline more sharply in our study than species with smaller geographic distributions. One possible explanation is that habitat degradation or destruction outside the study sites but in the local area has affected grassland, thicket, and woodland more so than old-growth remnant patches, resulting in more severe population declines for species found predominantly in regenerating vegetation types. In this scenario, the significance of range extent would be largely coincidental. However, range extent could conceivably be more directly impinging on local population trends. The magnitude of change in bird density in response to broad-scale environmental change is generally greatest at the edge of a species' range, and environmental change that negatively affects species tends to result in a contraction of the range towards the core [28]. Because our study site is on the Indian Ocean coast and relatively near the southern most point of the African continent, the forests are at the edge of the range of many species. Thus, the range extent variable generally reflects the distance between our study site and the central point of the range. It might be that change in abundance is not only greatest at the edge but also dependent on how far away the edge is from the core. However, whether the central point of the ranges of species in our analysis corresponds to core range requires further investigation, although there is some evidence that it should be so [36].

That species with predominant habitat in regenerating vegetation types tended to decline more than others did should not overshadow the conclusion that most birds, regardless of habitat affinity, have declined. Overall density of the 76 bird species assessed declined significantly at an alarming rate of 12.2% per year across old-growth coastal dune forests and woody regenerating vegetation types. Recent below average mean annual rainfall might be expected to affect most bird species via effects on survival and breeding success (see [31]) and thus, might have contributed to the widespread decline across species and vegetation types. If so, predictions of climate change induced rainfall reductions (see [37]) are concerning. It is also possible that the bird declines are merely temporary responses to drought. However, there are other possibilities that warrant further research including extinction debt [38], ecological traps or sinks [39], and broad-scale habitat change. Habitat destruction and environmental change at a macroecological scale could be affecting population trends at the local scale as reported elsewhere [28]. This hypothesis is in line with the importance of range extent in our analysis and implies that bird population declines are much more widespread across the region and perhaps the continent.

Severe and widespread declines of bird populations have been recorded throughout the world (*e.g.* [40]–[44]), and identification of these declines was largely the result of massive survey efforts in decades-long, nationwide programs such as the Breeding Bird Survey and Common Bird Census in the United Kingdom and the North American Breeding Bird Survey in the United States and Canada [40], [45]. That similar declines have not been identified in Africa might be due to a lack of monitoring data, though some studies report declines of single species or small groups of species (*e.g.* [46]–[48]), and the decline of migratory bird populations in Europe indicate potential problems in wintering grounds in Africa [49].

Other studies show that many forest bird species occur in human modified landscapes that appear, from a human perspective, quite different from undisturbed forest (*e.g.* [9], [15], [50]). Likewise, few species for which we assessed habitat affinities were strictly found in old-growth coastal dune forest while most were also found in woody regenerating vegetation. Thus, regenerating vegetation and remnant old-growth forests at our study site might provide valuable habitat for birds in a human modified coastal dune forest landscape. However, the decline of birds across our study site draws their persistence into question. While assessing population trends over time is a step towards understanding the processes that determine occurrence and persistence of birds in human modified landscapes, much more research is needed to elucidate the underlying mechanisms that generate trends—breeding success, survival, and dispersal.

In conclusion, remnant patches of old-growth forest and sites regenerating after mining in a human modified coastal dune forest landscape might provide valuable habitat for birds. Persistence of these bird communities might contribute to conservation not only of birds but also forests by enhancing functional connectivity between coastal forests in protected areas and other remnant patches through seed dispersal and pollination. However, further assessment of long-term monitoring data revealed population declines of most bird species assessed and a consistent reduction in bird density across vegetation types. Birds are sensitive to a host of ecological threats (see [44]) including habitat degradation [51] and fragmentation [52], invasive species [53], [54], climate change [25], [55], [56], emergent disease [57], [58], and pollution [59], [60], so bird declines identified here are a warning of environmental problems. Probable loss of valuable ecosystem services such as pollination, seed dispersal, and nutrient recycling with bird declines are also worrying [35] and might even threaten the coastal dune forest rehabilitation program which relies on processes of natural succession [10], [11]. More research is urgently needed to elucidate the mechanisms driving the decline and to assess whether declines are a local phenomenon or are also occurring at a broader geographical scale.

## Supporting Information

Table S1 **Transects per site per year in regenerating and old-growth sites.**
(DOC)

Table S2 **Relatively rare species.**
(DOC)

Table S3 **AIC selected detection function models for species pools.**
(DOC)

Table S4 **AIC model selection for validating species pooling assumption.**
(DOC)

Table S5 **AIC selected detection function models stratified by vegetation type (76 species pooled).**
(DOC)

Figure S1 **Change in rainfall over time.** Bars represent residual mean annual rainfall from the long-term (1977–2009) mean in mm.
(TIF)
