# Supplementary material for: Decline of Birds in a Human Modified Coastal Dune Forest Landscape in South Africa
Source: PLoS One. 2011 Jan 13;6(1):e16176. doi: 10.1371/journal.pone.0016176 (PMC3020955; doi:10.1371/journal.pone.0016176)
Supplement: Table S1 — Transects per site per year in regenerating and old-growth sites. (DOC) [file pone.0016176.s001.doc]

**Table S1.** **Transects per site per year in regenerating and old-growth sites.**

| Year | Observer | Transects per site | | | | | | | | | | |
| --- | --- | --- | --- | --- | --- | --- | --- | --- | --- | --- | --- | --- |
|  |  | Reg1 (32) | Reg2  (29) | Reg3  (25) | Reg4  (21) | Reg5  (17) | Reg6  (13) | Reg7  (9) | Reg8 (6) | Reg9 (3) | OG1 | OG2 |
| 1997 | A | 2^a^ | 2^a^ | 2^a^ | 2^a^ | 1^a^ | 4^a^ | - | - | - | 1^a^ | 2^a^ |
| 1998 | A | 4^a^ | 4^a^ | 4^a^ | 4^a^ | 4^a^ | 3^b^ | - | - | - | 4^a^ | 8^a^ |
| 2000 | A | 4^a^ | 4^a^ | 4^a^ | 4^a^ | 4^a^ | 3^b^ | - | - | - | 4^a^ | 4^a^ |
| 2001 | A | 4^a^ | 4^a^ | 4^a^ | 4^a^ | 4^a^ | 2^b^ | - | - | - | 4^a^ | 10^a^ |
| 2004 | B | 2^c^ | 3^c^ | 2^c^ | 2^c^ | 3^c^ | 2^c^ | 3^c^ | 3^c^ | - | - | 5^c^ |
| 2006 | C | 4^c^ | 5^c^ | 4^c^ | 4^c^ | 5^c^ | 4^c^ | 5^c^ | - | - | 5^c^ | 10^c^ |
| 2007 | D&E | 6^c^ | 7^c^ | 7^c^ | 11^c^ | 4^c^ | 4^c^ | 7^c^ | 6^c^ | 3^c^ | 11^c^ | 9^c^ |
| 2008 | D&E | 4^c^ | 5^c^ | 4^c^ | 4^c^ | 6^c^ | 7^c^ | 6^c^ | 4^c^ | - | 8^c^ | 6^c^ |
| 2009 | D&E | 6^c^ | 6^c^ | 6^c^ | 4^c^ | 5^c^ | 6^c^ | 5^c^ | 4^c^ | 9^c^ | 4^c^ | 8 ^c^ |

RegX sites are regenerating after mining—numbers in parentheses represent the regeneration age since mining as of 2009. OG sites are old-growth forests. ^a^ indicates transect length of 250 m,  ^b^ indicates transects length of 500 m, and ^c^ indicates transect length of 300 m.
