# Supplementary material for: Decline of Birds in a Human Modified Coastal Dune Forest Landscape in South Africa
Source: PLoS One. 2011 Jan 13;6(1):e16176. doi: 10.1371/journal.pone.0016176 (PMC3020955; doi:10.1371/journal.pone.0016176)
Supplement: Table S2 — Relatively rare species. (DOC) [file pone.0016176.s002.doc]

**Table S2**. Relatively rare species

| Common name | Scientific name |
| --- | --- |
| Bearded Scrub-Robin | *Cercotrichas quadrivirgata* |
| African Dusky Flycatcher | *Muscicapa adusta* |
| African Emerald Cuckoo | *Chrysococcyx Cupreus* |
| African Green-Pigeon | *Treron calvus* |
| African Paradise-Flycatcher | *Terpsiphone viridis* |
| African Pygmy-Kingfisher | *Ispidina picta* |
| Bar-throated Apalis | *Apalis thoracica* |
| Brimstone Canary | *Crithagra sulphurata* |
| Broad-billed Roller | *Eurystomus glaucurus* |
| Buff-spotted Flufftail | *Sarothrura elegans* |
| Cape Batis | *Batis capensis* |
| Cape Turtle-Dove | *Streptopelia capicola* |
| Cardinal Woodpecker | *Dendropicos fuscescens* |
| Chorister Robin-Chat | *Cossypha dichroa* |
| Common Cuckoo | *Cuculus canorus* |
| Crested Guineafowl | *Guttera edouardi* |
| Crowned Hornbill | *Tockus alboterminatus* |
| Eastern Bronze-naped Pigeon | *Columba delegorguei* |
| Eurasian Golden Oriole | *Oriolus oriolus* |
| Garden Warbler | *Sylvia borin* |
| Giant Kingfisher | *Megaceryle maxima* |
| Gorgeous Bush-Shrike | *Telophorus viridis* |
| Green Twinspot | *Mandingoa nitidula* |
| Grey Waxbill | *Estrilda perreini* |
| Icterine Warbler | *Hippolais icterina* |
| Klaas's Cuckoo | *Chrysococcyx klaas* |
| Lesser Honeyguide | *Indicator minor* |
| Long-billed Crombec | *Sylvietta rufescens* |
| Narina Trogon | *Apaloderma narina* |
| Neddicky | *Cisticola fulvicapilla* |
| Purple-banded Sunbird | *Cinnyris bifasciatus* |
| Purple-crested Turaco | *Gallirex porphyreolophus* |
| Red-chested Cuckoo | *Cuculus solitarius* |
| Red-fronted Tinkerbird | *Pogoniulus pusillus* |
| Southern Black Flycatcher | *Melaenornis pammelaina* |
| Spectacled Weaver | *Ploceus ocularis* |
| Spotted Flycatcher | *Muscicapa striata* |
| Swee Waxbill | *Coccopygia melanotis* |
| Woodwards' Batis | *Batis fratrum* |

Species names follow [29].
