# Supplementary material for: Decline of Birds in a Human Modified Coastal Dune Forest Landscape in South Africa
Source: PLoS One. 2011 Jan 13;6(1):e16176. doi: 10.1371/journal.pone.0016176 (PMC3020955; doi:10.1371/journal.pone.0016176)
Supplement: Table S3 — AIC selected detection function models for species pools. (DOC) [file pone.0016176.s003.doc]

**Table S3. AIC selected detection function models for species pools**

| Grouping | Model details | 1997 | 1998 | 2000 | 2001 | 2004 | 2006 | 2007 | 2008 | 2009 |
| --- | --- | --- | --- | --- | --- | --- | --- | --- | --- | --- |
| Pool A | $\hat{P}$*_a,t_* | 0.208 | 0.332 | 0.482 | 0.357 | 0.254 | 0.435 | 0.255 | 0.225 | 0.198 |
|  | SE | 0.018 | 0.017 | 0.027 | 0.017 | 0.039 | 0.018 | 0.019 | 0.023 | 0.080 |
|  | Best Model | HN+V | HN+V | HR | HN+V | HN | HR | HR+O | HR | HR |
|  | *L_t_* (m) | 4000 | 9500 | 8500 | 9500 | 7500 | 13800 | 22500 | 16200 | 17400 |
|  | *w* (m) | 40 | 40 | 40 | 40 | 40 | 30 | 40 | 40 | 40 |
| Pool B | $\hat{P}$*_a,t_* | 0.260 | 0.509 | 0.438 | 0.488 | 0.339 | 0.643 | 0.359 | 0.408 | 0.359 |
|  | SE | 0.016 | 0.018 | 0.016 | 0.019 | 0.040 | 0.040 | 0.014 | 0.023 | 0.025 |
|  | Best Model | HN | HR+V | HN+V | HR+V | HN | HR | HR | HN+O | HR |
|  | *L_t_* (m) | 4000 | 9500 | 8500 | 9500 | 7500 | 13800 | 22500 | 16200 | 17400 |
|  | *w* (m) | 40 | 40 | 40 | 40 | 40 | 30 | 40 | 40 | 40 |
| Pool C | $\hat{P}$*_a,t_* | 0.324 | 0.512 | 0.508 | 0.501 | 0.311 | 0.670 | 0.275 | 0.299 | 0.364 |
|  | SE | 0.028 | 0.021 | 0.021 | 0.020 | 0.030 | 0.044 | 0.012 | 0.029 | 0.025 |
|  | Best Model | HR | HR+V | HR+V | HN+V | HN+V | HR | HN+O+V | HR+O+V | HN+O+V |
|  | *L_t_* (m) | 4000 | 9500 | 8500 | 9500 | 7500 | 13800 | 22500 | 16200 | 17400 |
|  | *w* (m) | 40 | 50 | 50 | 50 | 50 | 30 | 50 | 50 | 50 |

See (Table 1) for species pool composition. Pool A comprises furtive species, Pool B intermediate, and Pool C conspicuous. Model details are described by $\hat{P}$*_a,t_*, the estimated mean probability of detection for species in the covered region *a* in year *t*; its SE; *L_t_,* the line length surveyed at time *t*; *w_t_*, the truncation distance; and the model key function and covariates. Model abbreviations as follows: “HR” for hazard-rate key, “HN” for half-normal key, “+V” for vegetation type as factor covariate, and “+O” for observer as factor covariate.
