# Supplementary material for: Decline of Birds in a Human Modified Coastal Dune Forest Landscape in South Africa
Source: PLoS One. 2011 Jan 13;6(1):e16176. doi: 10.1371/journal.pone.0016176 (PMC3020955; doi:10.1371/journal.pone.0016176)
Supplement: Table S4 — AIC model selection for validating species pooling assumption. (DOC) [file pone.0016176.s004.doc]

**Table S4.** **AIC model selection for validating species pooling assumption**

|  | ΔAIC | | |
| --- | --- | --- | --- |
| Model | Pool A | Pool B | Pool C |
| HN | 143.59 | 48.7 | 117.96 |
| HN + V | 102.38 | 5.47 | 48.17 |
| HN+O | - | 0 | 0 |
| HN+S | 138 | 55.38 | 90.84 |
| HR | 129.73 | 62.05 | 116.75 |
| HR + V | 133.45 | 2.61 | 145.27 |
| HR+O | 0 | 6.21 | 143.55 |
| HR+S | 141.16 | 72.21 | 171.47 |

ΔAIC=0 indicates the most supported model of the detection function for each species pool over the study period (years pooled with 1997 and 2006 excluded due to constraint in setting reasonable cutpoints). See (Table 1) for species pool composition. Pool A comprises furtive species, Pool B intermediate, and Pool C conspicuous. Model abbreviations as follows: “HR” for hazard-rate key, “HN” for half-normal key, “+V” for vegetation type as factor covariate, “+O” for observer as factor covariate, and “+S” for species as factor covariate. Models including species as a factor covariate had the highest ΔAIC in support of our species pooling assumptions. The half-normal key model with observer as a factor covariate failed to converge for Pool A.
