# Supplementary material for: Decline of Birds in a Human Modified Coastal Dune Forest Landscape in South Africa
Source: PLoS One. 2011 Jan 13;6(1):e16176. doi: 10.1371/journal.pone.0016176 (PMC3020955; doi:10.1371/journal.pone.0016176)
Supplement: Table S5 — AIC selected detection function models stratified by vegetation type (76 species pooled). (DOC) [file pone.0016176.s005.doc]

**Table S5. AIC selected detection function models stratified by vegetation type (76 species pooled)**

| Grouping | Model Details | 1997 | 1998 | 2000 | 2001 | 2004 | 2006 | 2007 | 2008 | 2009 |
| --- | --- | --- | --- | --- | --- | --- | --- | --- | --- | --- |
|  | Best Model | HR | HR | HR | HR | HR | HR | HN | HR | HN+O |
|  | *w* (m) | 40 | 40 | 40 | 40 | 40 | 30 | 40 | 40 | 40 |
| Old-growth | $\hat{P}$*_a,t_* | 0.270 | 0.467 | 0.571 | 0.465 | 0.380 | 0.579 | 0.335 | 0.430 | 0.382 |
|  | SE | 0.016 | 0.022 | 0.025 | 0.019 | 0.039 | 0.030 | 0.015 | 0.030 | 0.030 |
|  | *L_t_* (m) | 750 | 3000 | 2000 | 3500 | 1500 | 4500 | 6000 | 4200 | 3600 |
| Late woodland | $\hat{P}$*_a,t_* |  | 0.728 | 0.417 | 0.751 | 0.162 | 0.573 | 0.394 | 0.643 | 0.588 |
|  | SE |  | 0.047 | 0.028 | 0.040 | 0.298 | 0.035 | 0.017 | 0.057 | 0.028 |
|  | *L_t_* (m) |  | 1000 | 1000 | 2000 | 1500 | 3900 | 6000 | 3900 | 6600 |
| Early woodland | $\hat{P}$*_a,t_* | 0.346 | 0.674 | 0.787 | 0.676 | 0.450 | 0.712 | 0.348 | 0.388 | 0.614 |
|  | SE | 0.021 | 0.029 | 0.037 | 0.045 | 0.080 | 0.079 | 0.022 | 0.064 | 0.086 |
|  | *L_t_* (m) | 1500 | 2000 | 2000 | 2000 | 1200 | 2700 | 4500 | 3000 | 1500 |
| Thicket | $\hat{P}$*_a,t_* | 0.213 | 0.355 | 0.581 | 0.408 | 0.463 | 0.142 | 0.429 | 0.361 | 0.360 |
|  | SE | 0.034 | 0.024 | 0.029 | 0.030 | 0.082 | 0.438 | 0.050 | 0.050 | 0.031 |
|  | *L_t_* (m) | 500 | 2000 | 2000 | 1000 | 1500 | 2700 | 3300 | 3900 | 4500 |

Model details are described by $\hat{P}$*_a,t_*, the estimated mean probability of detection for species in the covered region *a* in year *t*; its SE; *L_t_,* the line length surveyed at time *t*; *w_t_*, the truncation distance; and the model key function and covariates. Model abbreviations as follows: “HR” for hazard-rate key, “HN” for half-normal key, and “+O” for observer as factor covariate.
