# Supplementary figures and images for: Decline of Birds in a Human Modified Coastal Dune Forest Landscape in South Africa
Source: PLoS One. 2011 Jan 13;6(1):e16176. doi: 10.1371/journal.pone.0016176 (PMC3020955; doi:10.1371/journal.pone.0016176)

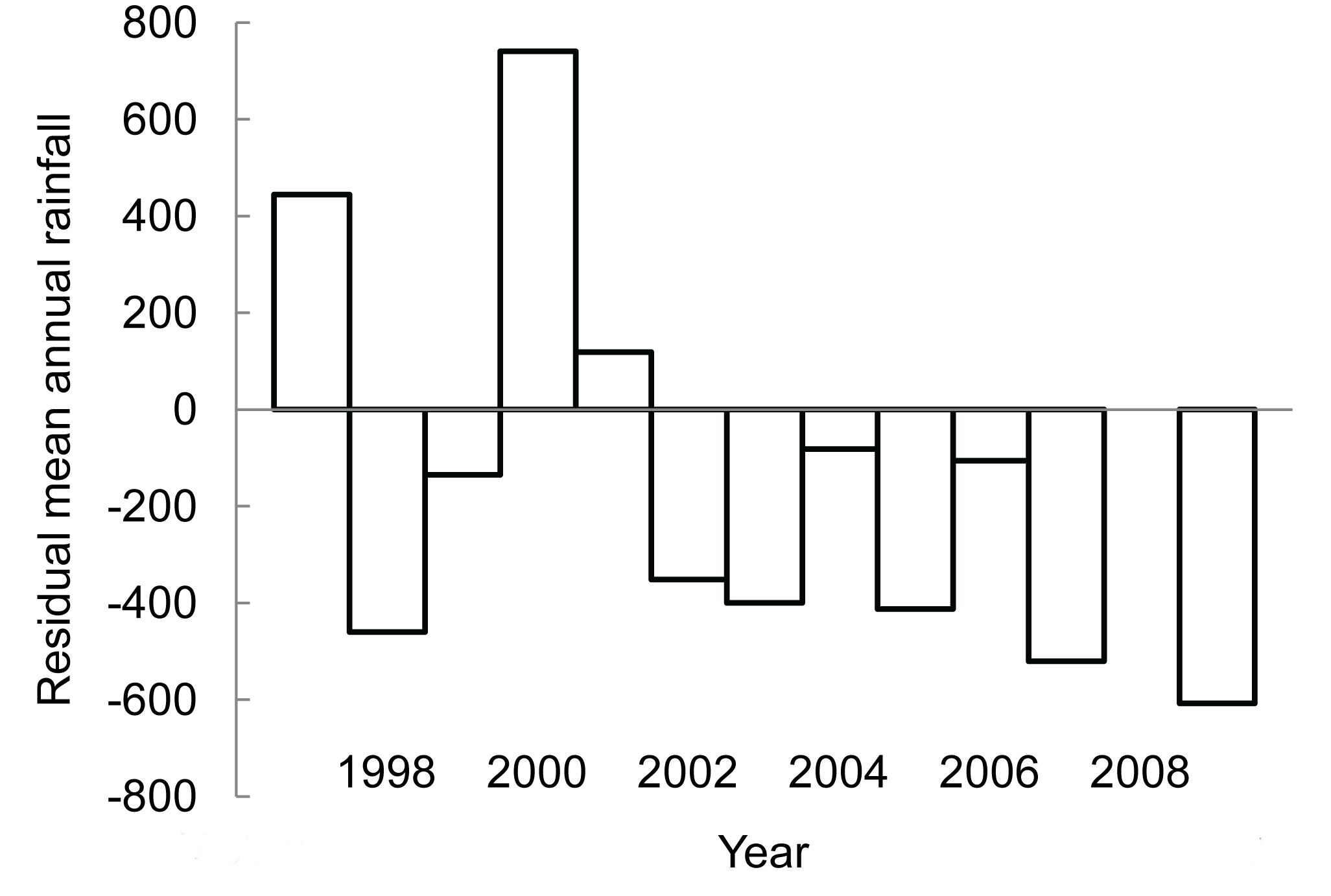

Supplement: Figure S1 — Change in rainfall over time. Bars represent residual mean annual rainfall from the long-term (1977–2009) mean in mm. (TIF) [file pone.0016176.s006.tif]
